# National outbreak of *Yersinia enterocolitica* infections in military and civilian populations associated with consumption of mixed salad, Norway, 2014

**DOI:** 10.2807/1560-7917.ES.2016.21.34.30321

**Published:** 2016-08-25

**Authors:** Emily MacDonald, Margot Einöder-Moreno, Katrine Borgen, Lin Thorstensen Brandal, Lore Diab, Øivind Fossli, Bernardo Guzman Herrador, Ammar Ali Hassan, Gro S Johannessen, Eva Jeanette Johansen, Roger Jørgensen Kimo, Tore Lier, Bjørn Leif Paulsen, Rodica Popescu, Charlotte Tokle Schytte, Kristin Sæbø Pettersen, Line Vold, Øyvind Ørmen, Astrid Louise Wester, Marit Wiklund, Karin Nygård

**Affiliations:** 1 Department of Infectious Disease Epidemiology, Norwegian Institute of Public Health, Oslo, Norway; 2 European Programme for Intervention Epidemiology Training (EPIET), European Centre for Disease Control and Prevention (ECDC), Stockholm, Sweden; 3 Department of Foodborne Infections, Norwegian Institute of Public Health, Oslo, Norway; 4 Norwegian Armed Forces, Norway; 5 District Office of Midt-Troms, Norwegian Food Safety Authority, Finnsnes, Norway; 6 District Office of Tromsø, Norwegian Food Safety Authority, Tromsø, Norway; 7 Norwegian Veterinary Institute, Oslo, Norway; 8 Department of Microbiology and Infection Control, University Hospital of North Norway, Tromsø, Norway; 9 Regional Office for Troms and Finnmark, Norwegian Food Safety Authority, Kautokeino, Norway

**Keywords:** Food-borne diseases, disease outbreaks, Yersinia enterocolitica, Norway

## Abstract

In May 2014, a cluster of *Yersinia enterocolitica* (YE) O9 infections was reported from a military base in northern Norway. Concurrently, an increase in YE infections in civilians was observed in the Norwegian Surveillance System for Communicable Diseases. We investigated to ascertain the extent of the outbreak and identify the source in order to implement control measures. A case was defined as a person with laboratory-confirmed YE O9 infection with the outbreak multilocus variable-number tandem repeat analysis (MLVA)-profile (5-6-9-8-9-9). We conducted a case–control study in the military setting and calculated odds ratios (OR) using logistic regression. Traceback investigations were conducted to identify common suppliers and products in commercial kitchens frequented by cases. By 28 May, we identified 133 cases, of which 117 were linked to four military bases and 16 were civilians from geographically dispersed counties. Among foods consumed by cases, multivariable analysis pointed to mixed salad as a potential source of illness (OR 10.26; 95% confidence interval (CI): 0.85–123.57). The four military bases and cafeterias visited by 14/16 civilian cases received iceberg lettuce or radicchio rosso from the same supplier. Secondary transmission cannot be eliminated as a source of infection in the military camps. The most likely source of the outbreak was salad mix containing imported radicchio rosso, due to its long shelf life. This outbreak is a reminder that fresh produce should not be discounted as a vehicle in prolonged outbreaks and that improvements are still required in the production and processing of fresh salad products.

## Introduction

*Yersinia enterocolitica* (YE) infection is the fourth most commonly reported cause of bacterial diarrhoeal disease in Norway [1]. Yersiniosis is notifiable to the Norwegian Institute of Public Health (NIPH) via the Norwegian Surveillance System for Communicable Diseases (MSIS). Since 2008, between 40 and 60 cases have been reported annually. More than 80% of yersiniosis cases in Norway are due to serotype O3, which is also the dominant cause of yersiniosis in Canada, Europe, Japan, and parts of the United States [2].The highest isolation rates have been reported during the cold season in temperate climates, including northern Europe and especially Scandinavia. The incubation period is generally under 10 days, but most often between three and seven days. Typical symptoms of yersiniosis include self-limiting acute febrile diarrhoea with abdominal pain, which can mimic appendicitis and has led to appendectomy [3]. YE infections have also been known to lead to sequelae such as reactive arthritis, erythema nodosum and conjunctivitis in up to 12% of cases [4].

Transmission most frequently occurs through eating contaminated food, particularly raw or undercooked pork, as the pig is the only animal consumed by humans which regularly harbours the pathogenic serovars O3 and O9 [2]. Case–control studies in Finland, Germany, New Zealand, Norway and Sweden have found that consumption of pork is associated with sporadic yersiniosis [5-9]. While outbreaks of yersiniosis have also been linked to consumption of pork [10,11], other food items such as milk, water and fresh vegetables have also been reported as a source of infection, and an outbreak of YE O9 due to imported ready-to-eat salad mix occurred in Norway in 2011 [11]. Most yersiniosis cases are sporadic and outbreaks are rarely reported [12]. Yersiniosis is rarely transmitted through sustained person-to-person transmission, although there have been previous outbreaks in which food handlers have been implicated [13].

### The event

On Thursday 8 May 2014, the Food Safety Authorities (FSA) District Office for Midt-Troms reported two cases of YE infections from a military base in northern Norway to the NIPH via the national web-based outbreak reporting system (Vesuv). Three additional cases were suspected at the time of the report. Concurrently, an increase of YE O9 infections was observed in MSIS with nine human isolates of YE O9 from geographically dispersed areas of the country received between 5 and 11 May 2014. The National Reference Laboratory (NRL) identified a common profile for the military and civilian cases through multilocus variable number tandem repeat analysis (MLVA), which had not been observed in Norway before this outbreak. In collaboration with the FSA and the military, an outbreak investigation was initiated to ascertain the extent of the outbreak, determine whether all cases were linked to the military and identify the source of the yersiniosis outbreak in Norway in order to implement control measures and prevent further spread.

## Methods

### Case finding

#### Outbreak case definition

For this outbreak a case was defined as any person with laboratory-confirmed YE O9 infection with the outbreak MLVA profile (5-6-9-8-9-9) with onset of symptom between 1 March and 15 June 2014. 

#### Case finding among civilians

In Norway, YE is reportable via MSIS and all isolates of presumptive YE are forwarded from clinical microbiology laboratories to the NRL where they are routinely characterised phenotypically, biotyped, tested for markers of plasmid-associated virulence factors and serogrouped against O3, O5,27, O8 and O9. Isolates can also be tested for a range of other serogroups if needed. The isolates are then MLVA-typed by the method described by Gierczyński et al. [14], locally adjusted to capillary electrophoresis.

#### Case finding on military bases

The Norwegian Armed Forces is a conscript military with 33 military bases throughout the country. Three military bases in the county of Troms in northern Norway (military bases T1, T2 and T3) and one military base in the county of Hedmark in south-eastern Norway (military base H1) reported cases to the NIPH. Base T1, the largest of the three bases, is located ca 40 km from base T2 and ca 30 km from base T3. The population of the military bases is composed primarily of privates, who are mostly Norwegians completing one year of mandatory military service. The soldiers belonging to each base are organised in companies, typically composed of 100 to 150 people. Bedrooms are typically shared by four to six people; bathrooms can be shared by up to 50 people. Privates and officers eat in the same mess halls, which are organised such that soldiers take food from a buffet table offering several hot and cold meal options, as well as a cold salad bar.

Information about cases on military bases was collected through the Military Health Officer. On 13 May the Military Health Office requested that all soldiers based at the three bases in Troms report to the healthcare centre if they had gastrointestinal symptoms, for isolation and testing. All cases diagnosed with yersiniosis were subsequently sent home from the military base until they provided a stool sample negative for YE. All kitchen staff on base T2 were tested, regardless of presence of symptoms, while kitchen staff from the other bases were only tested if symptomatic.

#### International enquiry

On 16 May the NIPH sent a message via the European Centre for Disease Prevention and Control (ECDC) Epidemic Intelligence Information System asking whether other European countries were also observing an increase in cases of YE infections.

### Investigating the source of infection

#### Trawling questionnaire and further development of a short questionnaire for civilian cases

The initial cases, both military and civilian, were interviewed using a standardised 22-page trawling questionnaire designed to generate hypotheses for possible sources of infection in a food-borne outbreak. For the identified military cases this questionnaire was administered on base by the local FSA, prior to being sent home from the base. For microbiologically-confirmed outbreak cases identified by the NRL that did not have any connections to a military base, the district FSA would visit the residence of the case to conduct the interview, as well as to collect food samples. The trawling questionnaire included detailed questions about food consumption and purchases, animal contact and environmental exposures in the week before onset of symptoms, as well as clinical and demographic information.

Subsequent to analysing information from the trawling interviews, a shorter questionnaire was developed for civilian cases. This questionnaire focused on foods of most interest, which included pork products and raw vegetables. It also included questions about potential locations of exposure, such as restaurants and cafeterias. The short questionnaire was administered to seven civilian cases through the FSA either by phone or in person.

#### Case–control investigation in the military setting

A case–control study was designed in order to identify the vehicle of infection among privates from two of the military bases. Cases identified by 29 May among privates in base T1 and among privates in base T2 were included in the study. Cases from the two most affected companies in base T2 were excluded *a priori* as additional factors affecting the occurrence of disease were suspected, including secondary transmission. Four controls were selected for each case, frequency matched by company. Due to security reasons, access to lists of privates belonging to each company was not provided to the investigators. Therefore, military officials from the relevant bases were given instructions to systematically select controls from an alphabetical list. In total, 21 cases (10 cases from T1 and 11 cases from T2) and 82 controls (44 controls from T1 and 38 controls from T2) were included in the case–control study.

Based on the hypotheses generated from the trawling questionnaire, a short self-administered questionnaire was developed for the case–control study. Menus from the military kitchens were available and used in this process. A total of 36 salad/vegetables items, 17 pork products and seven prepared salads that are served in the mess hall on a regular basis were included in the questionnaire, which was piloted with the head cook of the military kitchens and the brigade veterinarian before dissemination. Given the wide range in onset dates in cases and the anticipated difficulty for military personnel to remember the specific food items consumed from a buffet on specific days, both cases and controls were asked to indicate what food items they consume in a typical two week period in the mess hall.

#### Data collection

All controls for the case–control study were gathered in groups and interviewed in their respective military bases on 27 and 28 May 2014. Study participants were distributed the paper questionnaire which they were asked to complete. Photographs of different salad types were shown on a projector. Cases were interviewed by telephone by employees of the NIPH between 29 May and 10 June, as they had been asked to return home after being diagnosed and many had left the military base at the time of the study. Cases were sent an email with the same photographs of the salads shown to the controls and were asked to refer to the images while being interviewed.

#### Data analysis

Data were entered in the web-based questionnaire tool Questback. We calculated the number of people exposed to various food items, number of ill people among the exposed and unexposed and attack rates (AR) for all food items. We first analysed the association of each food item with yersiniosis one by one (univariable analysis). In the next step we selected food items which had odds ratios (OR) with a p-value lower than 0.25 and that had at least 50% of the cases exposed. Of these, we selected the three variables with lower p-value and stratified. Multivariable analysis was performed using logistic regression with OR, adjusted for military camp. We also calculated the dose-response association between the amount of salad consumed (never, once per month, once per week, several times per week and every day) and yersiniosis. This dose-response was also analysed for the amount of pork meat consumed. Descriptive analyses were performed in Excel and Stata 12, and univariable and multivariable analyses were performed in Stata 12.

#### Microbiological investigation of food samples

During site inspections, food samples were collected from the military base kitchens as well as from several commercial kitchens that had served civilian cases. Food samples were also collected from the homes of civilian cases. Samples were submitted to the Norwegian Veterinary Institute for analysis. The samples were analysed according to the ISO/WD Microbiology of food and animal feeding stuffs – Horizontal method for the detection of presumptive pathogenic YE (version 2012–12–01), which included direct plating and alkali treatment of both peptone-sorbitol-bile (PSB) and irgasan-ticarcillin-potassium chlorate (ITC) enrichment broths [15]. The samples were plated on both cefsulodin-irgasan-novobiocin (CIN) agar and a CHROMagar Yersinia enterocolitica (Paris, France). In addition, the samples were cold-enriched using a modified version of the Nordic Committee on Food Analysis method 117 (NMKL 117) [16]. The PSB enrichment broths and suspicious colonies were examined for the *ail* gene, an indicator for pathogenic YE, by polymerase chain reaction (PCR) [17].

### Traceback investigation

The FSA inspected the military base kitchens on 9 May, 13 May as well as 27 and 28 May 2014. A traceback investigation was conducted by the FSA on food items by reviewing documentation for suspected food products delivered to the military kitchens and commercial kitchens/cafeterias where civilian cases had eaten. The FSA contacted the distributers of suspected food items and conducted inspections where necessary.

## Results

### Description of the outbreak

**Figure 1:**
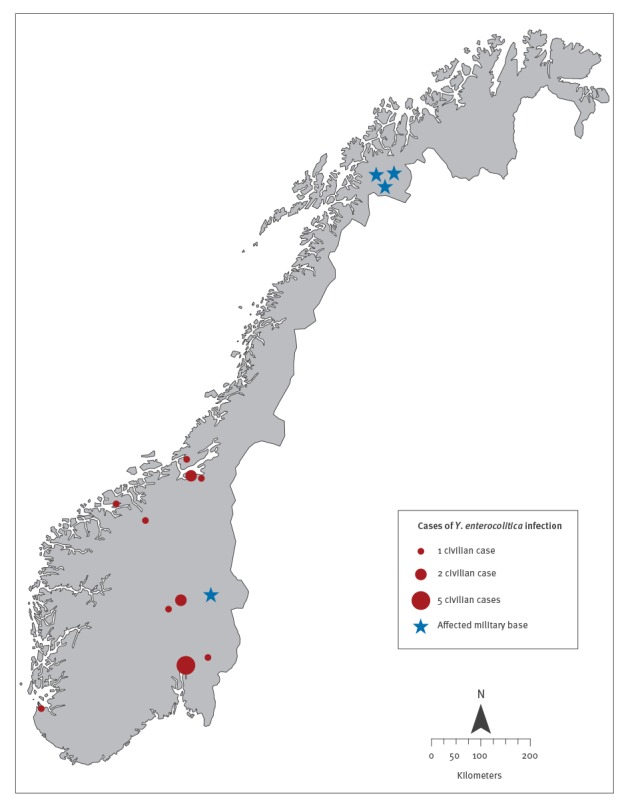
Geographical distribution of cases of *Yersinia enterocolitica* infection by military base and municipality of residence, Norway 2014

As of 29 July 2014, 133 confirmed cases of YE O9 infections were reported to the NIPH. Almost 90% of the confirmed cases (n = 117) had a confirmed link to one of four different military bases (Figure 1). Sixteen cases had no reported links to a military case. These cases resided in six different counties in Norway – Oslo (n = 5), Sør-Trøndelag (n = 4), Oppland (n = 3), Møre og Romsdal (n = 2), Akershus (n = 1), and Rogaland (n = 1). The 16 civilian cases ranged in age from 24 to 95 years (median: 39 years) and just over half were female (n = 9). 

Of the 117 cases from the military bases with identical MLVA profiles, almost all were reported from three bases in Troms county: T1 (n = 14), T2 (n = 88) and T3 (n = 3). Four cases were reported from military base H1 in Hedmark county and three cases had links to more than one military base (including either T2 or H1). For five cases the base was unknown. Cases linked to the military bases ranged in age from 19 to 57 years (median age: 21) and 21% were female (n = 24). Military cases belonged to at least seven battalions and fourteen companies. At least 32% (n = 37) of all military cases belonged to Company X of military base T2. Although the exact number of privates in the company is unknown, assuming a total company membership of between 100 and 150, the attack rate for Company X would have been between 25% and 37%. The company with the second highest attack rate, also from base T2, had 10 cases reported, corresponding to an attack rate of 7% to 10%. Seven kitchen personnel were diagnosed with yersiniosis, of which two were asymptomatic. All of these employees worked in base T2. Symptomatic kitchen staff were not identified at any of the other bases.

**Figure 2:**
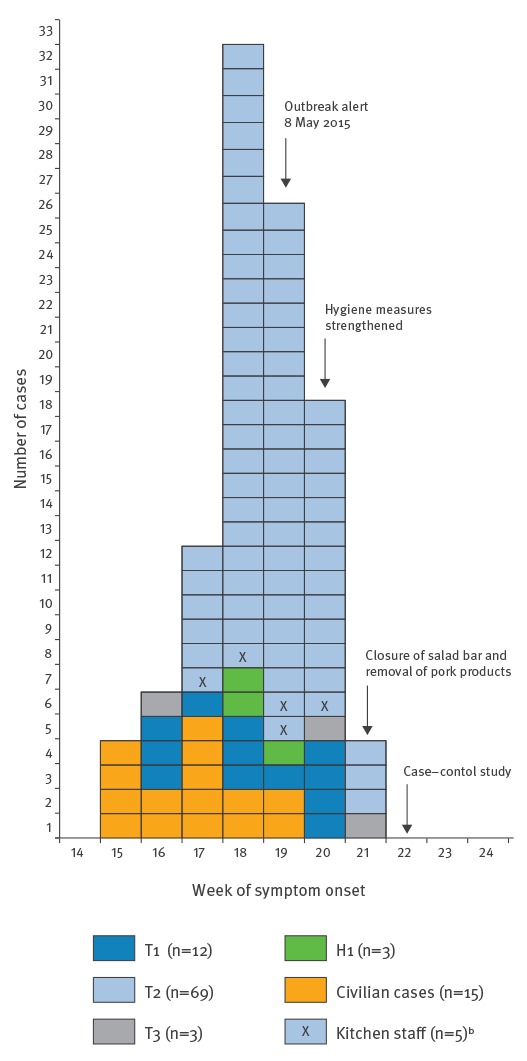
Distribution of cases of *Yersinia enterocolitica *infection by week of symptom onset, Norway 2014 (n=102)^a^

Symptom onset among cases with this information available (n=102) ranged from 9 April to 28 May 2014 (Week 15 to Week 21) (Figure 2). For civilian cases, most (n=12) had symptom onset from Week 15 to Week 17, while over 90% of military cases (n=81) had symptom onset between Week 17 and Week 20.

Week 14 starts on 31 March.

In the legend military bases where cases occurred are indicated by T1, T2, T4 (for bases in Troms county) and H1 (for a base in Hedmark county).

^a ^Of a total 133 outbreak cases, date of symptom onset was available for 102.

^b ^The five kitchen staff are included among the 69 cases in military base T2.

International requests for information produced no reports of similar yersiniosis outbreaks in other European countries.

### Investigating the source of the outbreak

#### Trawling interviews

Eighteen military cases, as well as nine of the total 16 civilian cases were interviewed using the hypothesis-generating questionnaire. The results of the trawling questionnaires from the military bases indicated that almost all soldiers ate all their meals in the same mess halls. The military mess halls offered a buffet, which meant that soldiers could choose what to take, but most cases were unaware of how food was prepared and which ingredients were used. Many cases reported consuming salad from the salad bar. The results of the trawling questionnaires for civilian cases suggested that all but one of the cases had eaten from restaurants or commercial kitchens. In particular, 12 of the 16 civilian cases interviewed reported eating from salad bars at workplace cafeterias.

#### Case–control study

In the case–control study, 10 food items had at least 50% of cases exposed and had a p-value < 0.25 in the univariable analysis (Table). These were included in the multivariable analysis.

**Table:** Univariable results of the case–control study of military bases T1 and T2, outbreak of *Yersinia enterocolitica* O9 infections, April–June 2014, Norway

Exposure	Cases (n = 21)		Controls (n = 82)		OR (95% CI)	P-value
	Total	Exposed N (%)	Total	Exposed N (%)		
Salad mix	21	20 (95)	82	57 (70)	8.77 (1.24–377.81)	0.015
Iceberg salad	21	20 (95)	82	62 (76)	6.45 (0.90–280.52)	0.046
Cooked ham	21	20 (95)	81	63 (78)	5.71 (0.79–249.76)	0.067
Onion	21	19 (91)	81	54 (67)	4.75 (1.01–44.52)	0.031
Arugula	21	11 (52)	82	20 (24)	3.41 (1.12–10.35)	0.013
Red salad leaves	21	11 (52)	81	20 (25)	3.36 (1.10–10.19)	0.014
Chopped ham	21	18 (86)	82	54 (66)	3.11 (0.80–17.71)	0.077
Salami	21	19 (91)	81	63 (78)	2.71 (0.56–26.03)	0.192
Roast beef	21	17 (81)	81	55 (68)	2.01 (0.57–8.97)	0.242
Cauliflower	21	11 (52)	81	54 (67)	0.55 (0.19–1.65)	0.225

CI: confidence interval; OR: odds ratio.

Of the 10 significant food items in univariable analysis, salad mix and arugula were the most likely to be associated with illness in multivariable analysis. Cases were 10 times more likely to have eaten salad mix than controls (OR:  10.26; 95% confidence interval (CI): 0.85–123.57, p-value: 0.067) and 95% of the cases (20/21) had eaten the salad mix compared with 70% of the controls (57/82). Cases were almost six times more likely to have eaten arugula than controls (OR: 5.48; 95%CI: 1.19–25.19, p-value: 0.029) and 52% of the cases (11/21) had eaten arugula compared with 24% of the controls (20/82). A dose-response relationship was observed between consumption of salad and illness. We observed that for every day salad was eaten, the risk increased by 9%.

#### Testing food samples

Fifteen food samples were taken from mess halls, cafeterias and private homes at the time of inspection on 27 May 2014. These included three samples of fresh-cut mixed salads, one shredded iceberg lettuce, six samples of other types of leafy greens (whole heads), one sample of carrots (snacks carrots), three samples of ham and one of bacon. The relevant batches of the consumed food products were already eaten or destroyed at the time of inspection. All food samples tested negative for pathogenic YE.

### Traceback investigation

Initial traceback investigations indicated that at least 14 of 16 civilian cases had eaten at kitchens or cafeterias that were supplied by the same distributor of fresh fruits and vegetables as the military kitchens in all four bases. This distributor holds ca 1% of the Norwegian market and uses only Norwegian produce during the summer, but imports 70–80% of produce during the winter months. At the time of the outbreak, the distributor had not yet begun using Norwegian products exclusively. Further investigation found that almost all kitchens could document receiving salad mix (which contains 80% iceberg lettuce and 20% radicchio rosso), whole iceberg lettuce or whole radicchio rosso with produce originating from one of two countries. Information about the imports of radicchio rosso showed that the import on 6 April 2014 came from the previous harvesting season, while the import on 16 April 2014 was from a new harvesting season. The ingredients were washed and salad mixes were assembled at a processing factory in Norway that belongs to a subsidiary company to the distributor. An inspection of the processing factory where the salad mixes were produced for the distributor found significant lapses in hygiene, including not changing water in rinsing tanks on a regular basis.

As control measures, the distributor improved hygiene measures, and the military bases thoroughly cleaned the bathrooms and kitchens, and increased awareness on hand hygiene among the soldiers. From Week 21, the kitchens in all three bases in Troms voluntarily elected to close the salad bar and refrain from serving pork products until the outbreak was resolved.

## Discussion

This outbreak of yersiniosis infection among civilians and members of the military was likely associated with consumption of fresh salad products. The geographically widespread occurrence of the yersiniosis cases and the prolonged duration indicated that the source of the product was widely distributed and available for a sustained period of time, which does not immediately suggest fresh produce as a source. However, the traceback investigations’ results for both the civilian and military cases strongly indicate that almost all cases were exposed to salad products supplied by the same distributer. Although the specific food item responsible for the outbreak could not be identified, the traceback investigation points towards one of two types of salad vegetables as the source: radicchio rosso and iceberg lettuce. Of these, radicchio rosso was considered to the be the most biologically plausible ingredient as it was the only salad component that keeps long enough to fit with the duration of this outbreak. Radicchio rosso has a shelf life of up to 150 days [18], while iceberg lettuce has a shelf life of less than two weeks. In addition, radicchio rosso is stored at +1 °C before it is supplied to the market. These storage conditions allow the growth of YE as this bacterium is able to grow down to –2 °C. If the implicated radicchio rosso was from the previous harvesting season and was imported before the changeover to Norwegian produce, it may have been stored for a long period of time, facilitating microbiological growth. Given the uncommon serotype and novel MLVA profile, it is suspected that the contamination of an imported salad product occurred outside of Norway, but potential lapses in processing after importation may have contributed to the spread.

All samples of salad products were negative for pathogenic *Yersinia*, but it is often challenging to isolate pathogenic YE from food samples. In a 2011 outbreak of yersiniosis associated with pre-mixed salad in Norway, non-pathogenic *Yersinia* was found in packaged salads [11], indicating that the long-term storage of this food product is conducive to the persistence of the bacterium. In this previous outbreak, non-pathogenic and environmental strains of *Yersinia*, including YE biotype 1A and *Y. kristensenii* were identified in samples of mixed salad and radicchio rosso. Radicchio rosso was considered to be the most likely source of contamination, given the microbiological results and the traceback investigation, although this could not be conclusively determined. In addition, several outbreaks of *Y. pseudoturberculosis* associated with consumption of vegetables such as carrots have also suggested the capacity for *Yersinia* bacteria to multiply in contaminated produce stored at cold temperatures [19-21]. Arugula is probably the most recognisable of the salads that were shown in the pictures, which may explain why it was significant in multivariable analysis. Salad mixes of the type we suspect to be implicated often contain arugula, although the traceback investigation indicates that arugula was not distributed to all the implicated commercial kitchens.

The case–control study in the military camps demonstrated that cases were 10 times more likely to have eaten salad mix than controls. Despite not reaching statistical significance, this result, along with other epidemiological evidence, excludes pork and supports salad as the likely source of infection. However, the findings do not allow for incrimination of a specific type of salad leaf. In the questionnaires we asked the respondents to indicate what they consume in a typical two week period rather than the two weeks before the onset of symptoms. While this supports that the cases eat all types of salads more frequently than controls, framing the questions in this way may have obscured an exposure that was specific to the period before the outbreak. In addition, as the salads were prepared in commercial kitchens, the study participants were not responsible for preparing the salads and may have been unable to discern the different types of salad. Although we tried to minimise this problem by showing photographs of different salad types to the study participants, many of the leaves have a similar appearance and cannot easily be distinguished.

Outbreaks of gastroenteritis at military bases of differing aetiologies, particularly norovirus, are not uncommon [22-27], but to our knowledge only one previous military outbreak of yersiniosis has been reported, from naval troops and infantry in Finland in 1973 [28]. Military bases present a unique opportunity for epidemiological investigation and for implementation of control measures as the population is well defined, attends the same healthcare facility and is responsive to requests to participate in investigations. The military may also have additional incentives to prevent and quickly control outbreaks that occur, as a matter of national security. Due to the communal living space, washrooms and kitchens, outbreaks in military camps can spread quickly through person-to-person transmission and the implementation of control measures can be difficult. In a recent study among military personnel deployed as part of the Ebola response in Sierra Leone, incidence of gastroenteritis was found to be lower than in military personnel deployed in Afghanistan [29]. Hygiene policies were similar in both contexts with the exception of hand washing, which occurred much more frequently in Sierra Leone. Although the deployment context is different than being on base, these results reinforce the importance of basic hygiene practices in reducing the spread of gastroenteritis in a military context. For these reasons, emphasis on personal hygiene, isolation of symptomatic cases and extensive disinfection of common areas, kitchens and washrooms are important measures to implement quickly. 

However, few accounts of person-to-person transmission of YE infection exist and are limited to exposures in nosocomial or family settings [30,31]. A study in Denmark on the occurrence of household outbreaks associated with different pathogens found that the tendency for YE to cause household outbreaks was low compared with other bacteria, like *Salmonella* Enteritidis and *Shigella sonnei* [32]. Although it was not possible to document the proportion of cases who may have been infected through person-to-person transmission, this transmission route may have propagated the outbreak. It is possible that different approaches to hygiene may have been taken by individuals and groups within specific companies, which may have explained the differences in attack rates in different companies. Varying approaches to testing among the leadership of different companies within the military or different levels of worry among some companies may have also led to increased interaction with the healthcare services. In any case, the importance of hand hygiene, safe food preparation measures and appropriate cleaning routines for washrooms during gastroenteritis outbreaks in military camps, regardless of the aetiology, cannot be ignored. 

The role of food handlers in this outbreak is unknown. As all seven positive food handlers worked in camp T2, this might explain the concentration of cases in that camp. Food handlers have been implicated in very few outbreaks of yersinosis. In 1981, investigators of an outbreak of YE O8 at a summer diet camp concluded that a food handler may have introduced the bacteria during food preparation [13]. In the 1973 yersiniosis outbreak in Finland, two civilian food handlers had positive faecal samples, but their role in the transmission was also unclear [28]. Concerning the military cases in Norway, infected food handlers may have propagated the outbreak by contaminating food that was served in the camp’s kitchen but it is unlikely that a food handler introduced the infection as the outbreak commenced simultaneously in several locations. In addition, the role of infected food handlers cannot explain the high attack rate among specific companies within camp T2. 

Other factors, including differences between companies in exposure to contaminated products, were also considered. As members of the same company tend to eat at the same time, it is possible that specific groups could have been more exposed to a contaminated batch depending on when they came to the mess hall or which batches were served to different companies. However, if this type of disproportionate exposure occurred, it cannot fully explain the prolonged duration of the outbreak.

## Conclusions and recommendations

This outbreak was the largest outbreak of YE infection in Norway as of 2014 and the first to be reported from a military context. The identification of the most likely source of infection, mixed salad, required combining information from the epidemiological, environmental, and traceback investigations from both civilian and military contexts. Person-to-person transmission may also have played a role in propagating the outbreak. The results of the investigation highlight that fresh produce should not be dismissed as possible sources in prolonged outbreaks. The implication of salad mix reinforces the need for improved control measures in the production chain for fresh produce.
